# EGFR-L861Q突变对TKI类药物敏感性预测分析及病例报道

**DOI:** 10.3779/j.issn.1009-3419.2015.09.11

**Published:** 2015-09-20

**Authors:** 星星 王, 雨桐 董, 婷婷 梁, 雄基 张, 克威 马, 永生 崔

**Affiliations:** 1 316000 舟山，舟山医院内科 Department of Medicine, Zhoushan Hospital, Zhoushan 316000, China; 2 132000 长春，吉林大学第一医院肿瘤中心 Department of Cancer Center, The First Hospital of Jilin University, Changchun 132000, China; 3 133000 延边，延边大学临床医学院 Clinical Medical College of Yanbian University, Yanbian 133000, China; 4 132000 长春，吉林大学第一医院肿瘤中心胸外科 Department of Thoracic Surgery, The First Hospital of Jilin University, Changchun 132000, China

**Keywords:** 肺肿瘤, EGFR-L861Q突变, 空间构象, 酪氨酸激酶抑制剂, Lung neoplasms, EGFR-L861Q mutation, Protein conformation, Tyrosine kinase inhibitors

## Abstract

**背景与目的:**

对于伴表皮生长因子受体（epidermal growth factor receptor, EGFR）敏感型突变的晚期非小细胞肺癌（non-small cell lung cancer, NSCLC）患者，小分子酪氨酸激酶抑制剂（tyrosine kinase inhibitor, TKI）的显著疗效众所周知。但对于晚期NSCLC伴EGFR-L861Q突变的患者，TKIs治疗是否敏感，治疗时机和治疗方案该如何选择，至今尚无确切的循证医学证据。本研究旨在通过分析EGFR-L861Q与敏感突变型EGFR-L858R及野生型EGFR蛋白质空间构象的差异，结合临床实例探讨晚期NSCLC伴EGFR-L861Q突变患者的最佳治疗方案。

**方法:**

利用同源模建重建野生型EGFR、敏感突变型EGFR-L858R及突变型EGFR-L861Q蛋白质的空间构象，并分析这三种空间构象之间的差异。

**结果:**

敏感突变型EGFR-L858R与野生型EGFR蛋白质的空间构象差异显著。突变型EGFR-L861Q与敏感突变型EGFR-L858R及野生型EGFR的蛋白质空间构象均不完全相同。在临床中，我们总结了1例晚期NSCLC伴EGFR-L861Q突变的患者，应用化疗作为一线治疗，当肿瘤不再缩小时，换用TKIs维持治疗，复查肺部计算机断层扫描（computed tomography, CT），肿瘤较前相比进一步缩小。

**结论:**

通过对突变型EGFR-L861Q的蛋白质空间构象进行分析比对，结合临床实例，对于晚期NSCLC伴EGFR-L861Q突变的患者，一线化疗后达到疾病控制时，换用TKIs维持治疗，可能获得令人满意的临床疗效。

## Abstract (en)

**Background and objective:**

The significant efficacy of tyrosine kinase inhibitors (TKIs) has been approved for advanced non-small cell lung cancer (NSCLC) patients with activating epidermal growth factor receptor (EGFR) mutations. No clear evidence exists that EGFR-L861Q is sensitive to TKIs, and the best treatment for NSCLC patients with EGFR-L861Q mutation is undetermined. This study aims to discuss the best treatment for advanced NSCLC patients with EGFR-L861Q mutation by analyzing the differences among the structures of wild-type EGFR, activating mutant EGFR-L858R, and EGFR-L861Q mutation.

**Method:**

The protein structures of wild-type EGFR were reconstructed. EGFR-L858R and EGFR-L861Q mutation were activated. The differences among the three kinds of protein conformation were analyzed using homologous modeling technique.

**Results:**

The structure of EGFR-L858R and wild-type EGFR exhibited notable distinctions. The structure of EGFR-L861Q mutation was different compared with wild-type EGFR and activating mutant EGFR-L858R protein conformations. NSCLC patients with EGFR-L861Q mutation were given chemotherapy as the first-line of therapy, and TKIs were applied to maintain treatment when the tumor is unchanged. Effect evaluation result was improved when the lung computed tomography lesions were reviewed.

**Conclusion:**

The analysis of the protein conformation of EGFR-L861Q mutation and the curative effect of chemotherapy with TKIs could help predict the sensitivity of EGFR-L861Q to TKIs. Combining the analysis with a clinical case, maintenance treatment with TKIs may achieve satisfactory curative effect in advanced NSCLC patients who have achieved disease control after first-line chemotherapy.

肺癌的发生率在恶性肿瘤中居于首位，其中非小细胞肺癌（non-small cell lung cancer, NSCLC）约占80%^[1, 2]^。腺癌是NSCLC中常见的病理类型，在肺腺癌患者中，伴表皮生长因子受体（epidermal growth factor receptor, *EGFR*）突变的患者约占15%-20%^[3, 4]^，而中国肺腺癌患者中，*EGFR*的突变率更高，约为31.6%-50.7%^[5, 6]^。*EGFR*突变主要见于酪氨酸激酶编码区外显子18-21之间，其中46%为外显子19的ELREA氨基酸序列缺失，35%-45%为外显子21的L858R突变^[7, 8]^，这两种突变是对小分子酪氨酸激酶抑制剂（tyrosine kinase inhibitors, TKIs）敏感的突变类型，伴敏感突变的NSCLC患者应用TKIs的临床有效率为62%-83%^[9-12]^。而外显子21的L861Q突变仅占*EGFR*突变的1.12%^[3]^，且对TKIs的敏感性相对较低^[3, 13, 14]^。在国际上，关于TKIs治疗晚期NSCLC伴EGFR-L861Q突变患者的案例鲜有报道^[15-20]^。化疗是NSCLC的传统治疗方法，TKIs是应用日益广泛的分子靶向药物。对于存在EGFR-L861Q突变的晚期肺癌患者，治疗方案该如何选择，至今仍有争议。伴EGFR-L861Q突变的NSCLC是否对TKIs敏感，可能与其空间构象的改变密切相关^[21]^。为研究EGFR-L861Q突变对TKIs的敏感性，我们重建野生型EGFR、敏感突变型EGFR-L858R及突变型EGFR-L861Q蛋白质的空间构象，分析不同空间构象对TKIs敏感性的差异。本文旨在探讨晚期NSCLC伴EGFR-L861Q突变患者的最佳治疗方案。

## 1 材料与方法

利用同源模建的方法构建并分析野生型EGFR、敏感突变型EGFR-L858R及突变型EGFR-L861Q蛋白质的空间构象。野生型EGFR蛋白质的空间构象来源于蛋白质数据库（Protein Data Bank, PDB）（ID: 2ITX）^[22]^。EGFR-L858R是2573位T突变为G，使相应位点的亮氨酸被精氨酸替代，EGFR-L861Q是2828位的T突变为A，使该位点的亮氨酸突变为谷氨酰胺）。我们应用Discovery Studio2.5中的Build mutants模块，将野生型EGFR蛋白质空间构象中与EGFR-L858R和EGFR-L861Q突变位点对应的氨基酸分别用精氨酸和谷氨酰胺替换，从而构建敏感突变型EGFR-L858R和突变型EGFR-L861Q蛋白质的空间构象。

## 2 结果

### 2.1 空间构象分析结果

#### 2.1.1 敏感突变型EGFR-L858R与野生型EGFR蛋白质的空间构象存在显著差异

本文中空间构象分析结果与2009年在*Clin Cancer Res*发表的研究^[13]^和2013年在*J Clin Oncol*发表的研究^[23]^结果一致。与野生型EGFR蛋白质空间构象相比，在EGFR-L858R蛋白质的空间构象中，L858R突变导致活化环上的858位亮氨酸被精氨酸所取代，使活化环的空间构象发生改变，从而影响了ATP磷酸结合环的空间构象，这种改变使ATP结合口袋的裂隙增大，从而使活性结合位点更易于接近。

#### 2.1.2 突变型EGFR-L861Q与野生型EGFR及敏感突变型EGFR-L858R的蛋白质空间构象均不完全相同

在EGFR-L861Q的蛋白质的空间构象中，L861Q突变导致活化环上的861位亮氨酸被谷氨酰胺所取代，使活化环的空间构象发生改变，从而影响了ATP磷酸结合环的空间构象。L861Q突变与L858R突变虽然都在活化环上，但两者的突变位点所在位置不同，突变后导致的氨基酸变化也不一致，所以L861Q突变对ATP结合口袋裂隙变化的影响程度也不一样。

### 2.2 临床病例分享

#### 2.2.1 患者资料

王XX，男，52岁，因体检发现肺部占位，双侧颈部淋巴结肿大，为进一步明确诊治就诊于我院。既往：无嗜烟、嗜酒史；无家族遗传史。入院查体：右侧颈部淋巴结活检术后包扎固定，双侧颈部可触及数枚肿大淋巴结，质韧，触之无疼痛感，边界不清，最大约2 cm×1 cm，余未见明显异常。标准体力状况评分（Easten Cooperative Oncology Group, ECOG）为1分，全面评估后，明确诊断为右肺肺癌（腺型，cT3N3M1，Ⅳ期）、骨转移癌（T10）。*EGFR*基因突变检测结果回报：*EGFR*基因第21号外显子检测到点突变（L861Q）（图 3）。

**1:**
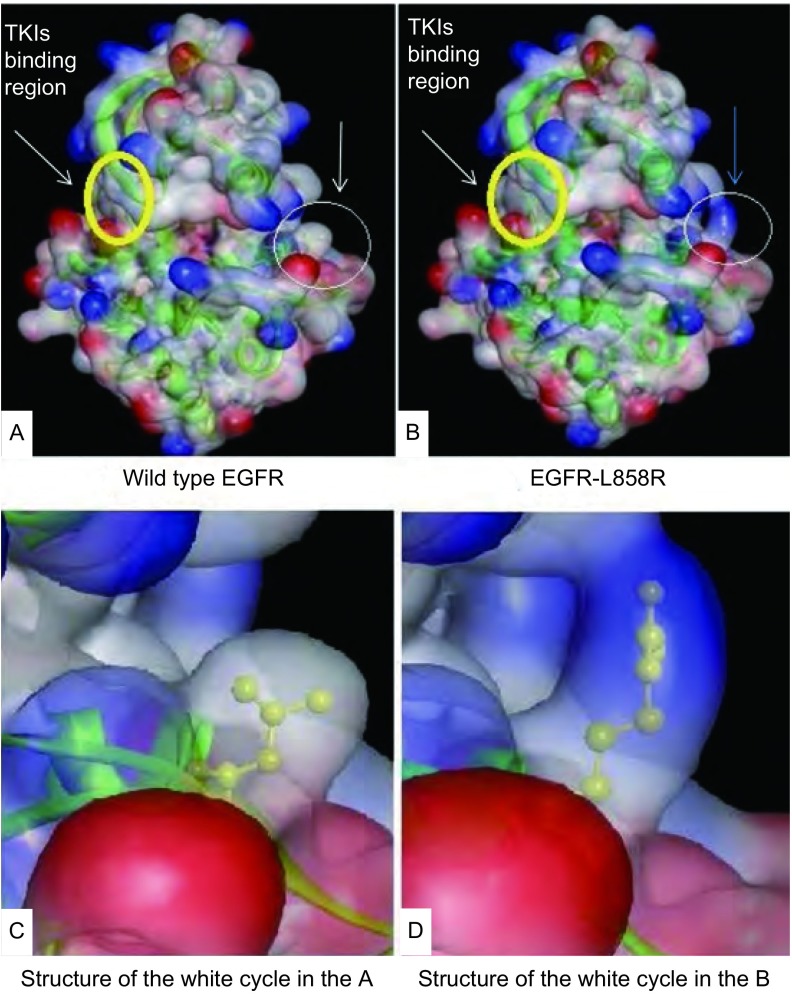
野生型EGFR和EGFR-L858R蛋白质空间构象。A：野生型EGFR蛋白质空间构象；B：突变型EGFR-L858R蛋白质空间构象，两者有明显差异；C、D：突变位点的局部空间结构；图中箭头所指的黄色圈内为TKIs结合区，白色圈内为突变位点的空间结构 The structure of wild type EGFR and activating mutant EGFR-L858R. A: The structure of wild type EGFR; B: The structure of activating mutant EGFR-L858R, they had notable distinction; C, D: The structure of mutation site. The yellow circle was the TKIs binding region, the white circle was the structure of the mutation site. TKI: tyrosine kinase inhibitor

**2:**
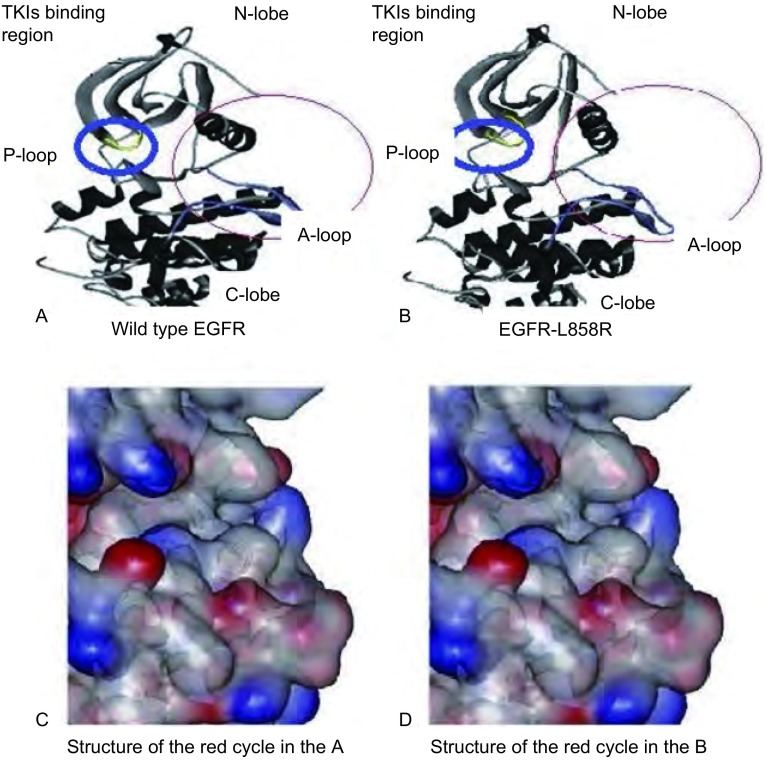
野生型EGFR和EGFR-L861Q蛋白质空间构象。A：野生型EGFR蛋白质空间构象；B：突变型EGFR-L861Q蛋白质空间构象，两者存在一定的差异；C、D：突变位点及周围的局部空间结构，图中箭头所指蓝色圈为TKIs结合区，箭头所指红色圈内为空间构象存在差异与否的部位。N-lobe为氨基端小叶，C-lobe为羟基端小叶，P-loop为ATP磷酸结合环，A-loop为活化环，是酪氨酸的活化中心 The structure of wild type EGFR and mutant EGFR-L861Q. A: The structure of wild type EGFR; B: The structure mutant EGFR-L861Q, they had some distinction; C, D: The structure of mutation site.The blue circle was the TKIs binding region, the red circle was the different part of the structure. N-lobe was amino-terminal leaflet, C-lobe was hydroxyl-terminal leaflet, P-loop was ATP phosphate binding loop, A-loop was activation loop, which was tyrosine activation center

**3:**
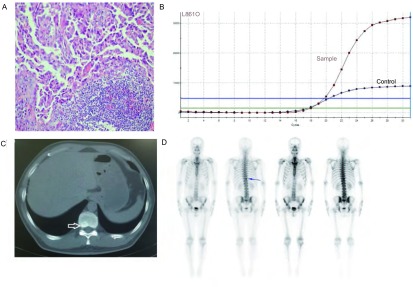
活检组织病理及基因突变检测结果。A：淋巴结活检结果：淋巴结转移性腺癌（HE染色，×200）；B：肿瘤组织基因检测结果：21号外显子点突变（L861Q）。样本信号曲线升起（呈S型曲线）表示该位点突变阳性；C：2013年4月14日肺CT，箭头所指为T10，显示T10骨转移部位；D：骨扫描结果，箭头所指为T10，显示放射性增高 Pathological result of lymph node biopsy and detection result of gene mutation. A: Pathological result of lymph node biopsy: Lymph node metastasis of adenocarcinoma; B: Exon 21 point mutation (L861Q). The sample signal curve rises (S-shaped curve) indicates that the mutation positive; C: Chest computed tomography scan of April 14, 2013, the arrow pointed to the position of bone metastasis of T10; D: The result of bone scan, the arrow pointed to the T10, which showed enhanced radioactivity

#### 2.2.2 治疗及疗效

治疗方案：患者分期为Ⅳ期，伴EGFR-L861Q突变，已无外科手术机会，首选治疗手段为化疗或分子靶向治疗。根据前期同源模建分析比对结果，我们首先选择培美曲塞联合顺铂（力比泰1, 000 mg d1+顺铂40 mg d2-d4）作为一线治疗方案，21 d为一个周期。每2周期进行疗效评价，疗效评价为持续部分缓解（partial response, PR）（图 4A-图 4C）。序贯应用分子靶向药物-盐酸埃克替尼维持治疗，30 d后复查肺计算机断层扫描（computed tomography, CT）（图 4D），肺部病灶大小较前（第4疗程后复查肺CT）进一步缩小（肺部病灶由2.3 cm×1.7 cm缩小至2.0 cm×1.5 cm）。后定期复查肺CT、脑CT、腹部CT及骨扫描提示病情稳定，2014年4月复查时发现疾病进展。

**4:**
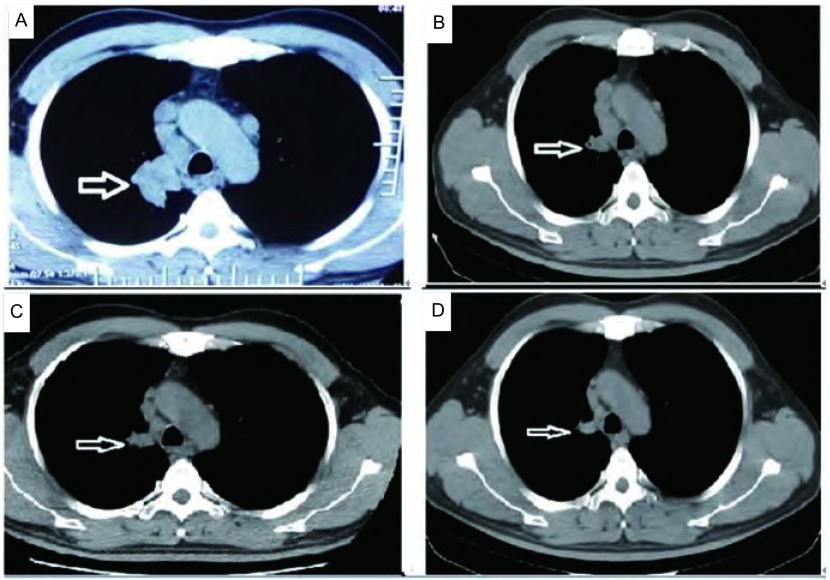
不同时期的肺部CT。A：治疗前肺部CT（2013年4月14日）；B：第2疗程化疗后肺部CT（2013年6月15日）；C：第4疗程化疗后肺部CT（2013年7月17日）；D：应用靶向药物维持治疗30天后肺部CT（2013年8月14日）。图中箭头所指为肺部肿瘤 The pulmonary computed tomography (CT) of different time. A: The pulmonary CT before treatment (April 14, 2013); B: The pulmonary CT after the second course of chemotherapy (June 5, 2013); C: The pulmonary CT after the fourth course of chemotherapy (July 17, 2013); D: The pulmonary CT after applying TKIs 30 days (August 14, 2013). The arrow pointed to pulmonary lesions

## 3 讨论

小分子靶向药物在肺癌治疗中的地位日益突显，EGFR已成为肺癌治疗的重要靶点^[24-26]^。Lynch等^[8]^和Paez等^[27]^首次报道，对于NSCLC患者EGFR酪氨酸激酶编码区基因突变状态是预测靶向药物疗效的一个必要前提条件。*EGFR*基因突变较常见于18-21号外显子区域，其中46%为外显子19的ELREA氨基酸序列缺失，35%-45%为21号外显子突变（L858R替代突变）^[7, 8]^，这两种突变均为敏感突变。对于伴敏感突变的亚裔NSCLC患者，应用TKIs作为一线治疗的疗效明显优于化疗^[28]^。

EGFR-L861Q突变仅占*EGFR*突变阳性的1.12%^[3]^，是由2828位点的T被A取代所致。临床上观察到：与NSCLC伴*EGFR*敏感突变位点的患者相比，NSCLC伴EGFR-L861Q突变患者对TKIs药物的敏感性相对较低^[3, 13, 14]^。Paez等^[27]^在*Science*杂志发表的实验结果指出，不同位点突变导致的空间构象改变与TKIs疗效密切相关，敏感突变对TKIs的敏感性要比野生型强100倍。这可能是因为空间构象与激酶活性密切相关，空间构象的改变影响了激酶的活性。既往Yun等^[29]^在*Cancer Cell*发表的研究提示，L861Q突变使861位的亮氨酸突变为谷氨酰胺，谷氨酰胺的一条侧链带有较大的电荷使其不易被EGFR-L861Q的非活性空间构象所容纳，而易被其活性构象所容纳。这种改变在一定程度上增加了酪氨酸激酶的活性，使TKIs易于与之结合，但与EGFR-L858R相比，其激酶活性的增加程度仍有较大差异。本文的空间构象进行分析结果显示，突变型EGFR-L861Q与野生型EGFR及敏感突变型EGFR-L858R的蛋白质空间构象均不完全相同。这提示EGFR-L861Q对TKIs的敏感性可能与EGFR-L858R不同，原因可能是L858R突变与L861Q突变虽然都在活化环上，但两者在活化环的位点不同，突变后导致的氨基酸变化也不同，所以突变后引起的空间构象变化也存在一定的差异。L861Q与L858R突变后引起空间构象的变化不同，对酪氨酸激酶活性的影响也不相同。2009年在*Clin Cancer Res*发表的研究^[13]^和在*J Clin Oncol*发表的研究^[23]^通过比较野生型EGFR和ATP类似物AMPPNP结合物与敏感突变型EGFR-L858R和AMPPNP结合物的空间构象，得出如下结论：与野生型EGFR-AMPPNP结合物的空间构象相比，EGFR-L858R-AMPPNP结合物的空间构象中ATP结合口袋的裂隙增大，这使活性结合位点更易于接近。这种改变可能是敏感突变型EGFR-L858R对TKIs敏感性较高的原因。但长期以来，由于NSCLC伴EGFR-L861Q突变的患者比较少见，对于该类患者，治疗方案该如何选择，仍无统一意见。

对于晚期伴EGFR-L861Q突变的NSCLC患者一线治疗该如何选择至今仍没有确切的循证医学证据。2014年欧洲临床肿瘤协会年会（European Society for Medical Oncology, ESMO）上也有研究提示伴EGFR-L861Q突变的NSCLC患者，应用TKIs的客观缓解率为39.6%，疾病控制率为75.5%，其疗效与应用化疗的疗效相近。因此，对于该例伴EGFR-L861Q突变的NSCLC患者，可以选择化疗作为一线治疗。

有研究^[30, 31]^证实对于晚期肿瘤患者，一线治疗后应用药物维持治疗能明显改善患者预后。对于伴EGFR-L861Q突变的晚期NSCLC患者应该选择什么药物用于维持治疗，至今仍有争议。2011年在美国芝加哥召开的美国临床肿瘤学会（American Society of Clinical Oncology, ASCO）会议上张力教授公布了关于晚期NSCLC一线化疗后维持治疗的INFORM研究结果，并于2012年在*Lancet Oncol*发表了相关研究^[31]^，该研究提示：基因突变患者应用吉非替尼维持治疗与应用安慰剂维持治疗的中位无进展生存期（progression free survival, PFS）存在明显差异（16.6个月*vs* 2.8个月，HR=0.17，*P*=0.006, 3），无基因突变患者吉非替尼组与安慰剂组的PFS无明显差异（2.7个月*vs* 1.5个月，HR=0.86），且该研究中的基因突变组包括敏感突变和少见突变。该研究结果提示对于存在*EGFR*突变的晚期NSCLC患者，一线化疗达到疾病控制后应用TKIs维持治疗是能够获益的。Giovannetti等^[32]^曾在分子药理学杂志发的研究提示，这种治疗模式能够增加疗效的原因可能与化疗后由于某种原因增加了细胞EGFR磷酸化水平而降低了Akt磷酸化水平有关。虽然有研究^[30, 33]^证实，对于晚期NSCLC患者，一线化疗病情控制后应用化疗药物维持治疗可以延长其PFS，但会增加血液毒性相关不良事件的发生率。因此，该例伴EGFR-L861Q突变的晚期NSCLC患者，一线化疗后达到疾病控制后，我们选用TKIs用于维持治疗，并获得了令人满意的临床疗效，其PFS达8个月，介于INFORM研究中一线化疗后应用靶向药物维持治疗的基因突变组和无基因突变组的PFS之间。这种治疗模式使患者获益的机制有待于进一步的基础研究。

既往研究显示空间构象对TKIs的疗效有一定的预测作用。本文构象分析结果显示少见突变型EGFR-L861Q与敏感突变型EGFR-L858R及野生型EGFR的蛋白质空间构象均不完全相同，提示伴EGFR-L861Q对TKIs的敏感性可能与野生型EGFR并不相同。2014年ESMO会议上有研究报道，伴EGFR-L861Q突变的NSCLC患者一线应用TKIs的疗效与化疗相近。文中报道的该例伴EGFR-L861Q突变的晚期NSCLC患者，当一线化疗4疗程后，肿瘤不再缩小时，应用TKIs维持治疗PFS达8个月，总体临床疗效尚令人满意。因此，对于晚期NSCLC伴EGFR-L861Q突变的患者，一线化疗后达到疾病控制时，改用TKIs维持治疗，可能获得令人满意的临床疗效。空间构象与TKIs的疗效可能有一定的相关性，而对于晚期NSCLC伴EGFR-L861Q突变患者的最佳治疗模式，有待于进一步临床观察及研究，我们期待更多、更大规模的临床研究数据以及更深入的、基础性的机制研究，使其更为明朗。
